# Systematic evaluation of supervised classifiers for fecal microbiota-based prediction of colorectal cancer

**DOI:** 10.18632/oncotarget.14488

**Published:** 2017-01-04

**Authors:** Luoyan Ai, Haiying Tian, Zhaofei Chen, Huimin Chen, Jie Xu, Jing-Yuan Fang

**Affiliations:** ^1^ Division of Gastroenterology and Hepatology, Shanghai Institute of Digestive Disease, Key Laboratory of Gastroenterology and Hepatology, Ministry of Health, State Key Laboratory for Oncogenes and Related Genes, Renji Hospital, School of Medicine, Shanghai Jiao-Tong University, Shanghai 200001, China

**Keywords:** gut microbiota, CRC, supervised classifier, prediction

## Abstract

Predicting colorectal cancer (CRC) based on fecal microbiota presents a promising method for non-invasive screening of CRC, but the optimization of classification models remains an unaddressed question. The purpose of this study was to systematically evaluate the effectiveness of different supervised machine-learning models in predicting CRC in two independent eastern and western populations. The structures of intestinal microflora in feces in Chinese population (*N* = 141) were determined by 454 FLX pyrosequencing, and different supervised classifiers were employed to predict CRC based on fecal microbiota operational taxonomic unit (OTUs). As a result, Bayes Net and Random Forest displayed higher accuracies than other algorithms in both populations, although Bayes Net was found with a lower false negative rate than that of Random Forest. Gut microbiota-based prediction was more accurate than the standard fecal occult blood test (FOBT), and the combination of both approaches further improved the prediction accuracy. Moreover, when unclassified OTUs were used as input, the BayesDMNB text algorithm achieved higher accuracy in the Chinese population (AUC=0.994). Taken together, our results suggest that Bayes Net classification model combined with unclassified OTUs may present an accurate method for predicting CRC based on the compositions of gut microbiota.

## INTRODUCTION

Colorectal cancer (CRC) ranks the third common malignancy and will cause over 600 000 deaths globally [1]. Screen detection and removal of precancerous lesions can largely prevent this cancer, as demonstrated by a declining incidence and mortality in countries with programmatic screening [2]. An ideal screening tool should have high sensitivity and specificity, with characteristics of noninvasiveness, feasibility, and affordability. To date, fecal occult blood testing (FOBT) and colonoscopy are the predominant screening tools [3–5]. Although colonoscopy has high sensitivity and could remove adenomas that may evolve to carcinoma, screening effectiveness is compromised by its invasiveness and cost. FOBT is the noninvasive standard screening method and has been shown to reduce cancer mortality [6–9]. However, blood in the stool is a nonspecific finding and because bleeding from cancers may be intermittent or simply not always detectable in a single sample of stool [10], it has limited sensitivity and specificity. Therefore, cancer incidence is affected only marginally [7, 9, 11, 12]. A more accurate noninvasive screening method is needed to identify those CRC patients as earlier as possible.

Recent progress in the relationship between gut microbiota and CRC opens new opportunities for developing novel strategies for CRC screening. High-throughput sequence technologies make it feasible to obtain a comprehensive view of the microbial ecosystem in gut microbiome and a quite different gut microbiome structure between CRC patients and healthy individuals has been reported by numerous studies [13, 14]. A number of bacterial species such as *Fusobacterium nucleatum* [15, 16], *Bacteroides fragilis* [17] and *Escherichia coli* [18] were involved in CRC carcinogenesis. The significant association between CRC and gut microbial composition allowed for non-invasive screening of CRC based on fecal microbiota as a promising method. Using Bayesian methods, Zackular first reported the possibility of the human gut microbiome as a screening tool of CRC [19]. While Zeller et al. arrived at the same conclusion that fecal microbiota would allow for accurate CRC screening, but using LASSO models [20]. A third model, Random Forest classifier, was also employed to test the diagnostic value of fecal microbiome for CRC, with the performance reached as high as 0.96 AUC [21]. Despite the increasing recognition of the CRC screening potential for fecal microbiome, the selection of classification models is diverse and remains an unaddressed question. It is also unclear if certain classifier model may have universal applicability in different populations and races.

Here, we systematically evaluated the performance of the supervised classifiers to predict CRC based on fecal microbiota. We recruited 141 participants and sequenced the V1∼V3 hypervariable regions of the 16S rRNA gene from the feces of each individual and employed different supervised machine learning algorithms to test their performance of predicting CRC based on fecal microbiota OTUs. We also validated our results in a French population.

## RESULTS

### The CRC prediction performance based on fecal microbiome varied among different classifiers in the Chinese population

**Figure 1:**
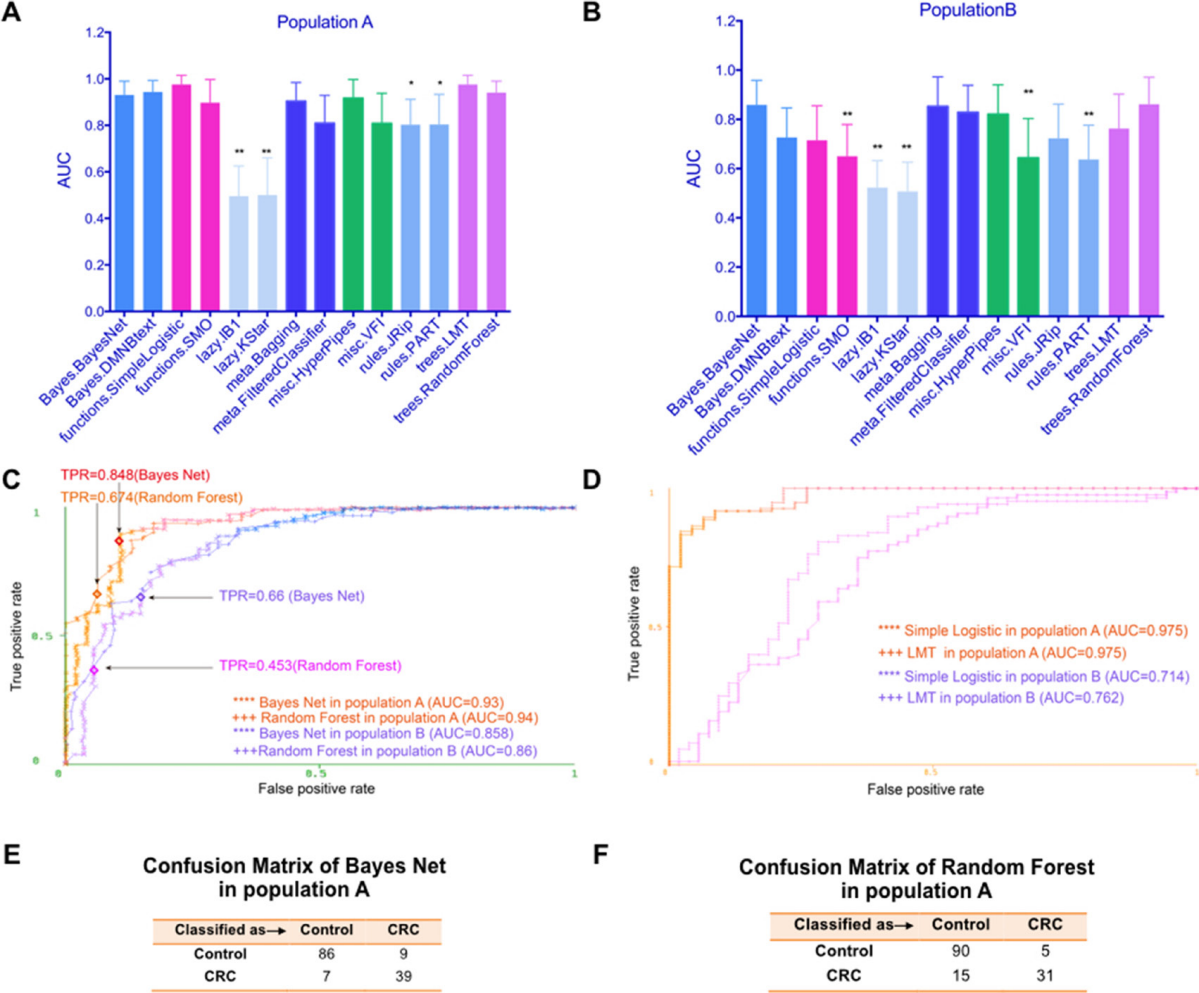
The CRC prediction performance based on fecal microbiome varied among different classifiers and populations (**A**) The test performance of different models using fecal microbiome on study population A was displayed using area under roc curve (AUC). (**B**) The test performance of different models using fecal microbiome on study population B was displayed using AUC. (**C**) ROC curves show the prediction ability of the Bayes Net and Random Forest models on study population A and B, respectively. TPR means true positive rate (sensitivity). (**D**) ROC curves of Simple Logistic and LMT algorithms on study population A and B. (**E**) Confusion Matrix of Bayes Net algorithm in population A. (**F**) Confusion Matrix of Random Forest algorithm in population A. *P < 0.05, **P < 0.01, the Bayes Net model was used as the control model.

To systematically assess the performance of different supervised machine learning algorithms in the realm of CRC detection based on fecal microbiome, we first imported the OTUs dataset in the species rank on the study population A (Table 1 for patient data) into the WEKA software. Features such as FOBT were removed. Only diagnosis and fecal microbiome OTUs information were included. We used the diagnosis feature as class. Then all available classifiers/algorithms such as Bayes Net [22, 23], Simple Logistic [24], JRip [25], J48 [26, 27], Random Forest [28], SMO [29] were applied to classify the samples (Supplementary Tables S1 and S2). As shown in Figure 1A, these algorithms showed different performance and some of them displayed high AUCs. For example, Simple Logistic and LMT algorithm [30] covered the maximum area under the curve (AUC=0.975, standard deviation (SD), 0.04), followed by Random Forest (AUC=0.94, SD, 0.05) and Bayesian methods (AUC=0.93, SD, 0.06), while lazy. IB1 model [31]only achieved an AUC of 0.693 (SD, 0.13). These results showed the classification ability among different models varied greatly, highlighting the necessity to choose a suitable one based on systematical approach.

**Table 1:** Summary of study population A and B

	Population A (Chinese)				Population B (French)			
	Control (*N* = 52)	CRA (*N* = 47)	CRC (*N* = 42)	*P*	Control (*N* = 61)	CRA (*N* = 27)	CRC (*N* = 53)	*P*
Age, years (mean ± SD)	52.29 ± 1.53	58.89 ± 1.48	62.88 ± 1.50	< 0.0001	60.57 ± 1.46	60.30 ± 1.67	66.81 ± 1.494	0.004
Gender (*n*,%)				0.544				0.190
Male	21(40%)	24(51%)	18(43%)		28(46%)	18(67%)	29(55%)	
Female	31(60%)	23(49%)	24(57%)		33(54%)	9 (33%)	24(45%)	
Positive FOBT (*n*,%)	7 (14%)	5 (11%)	16(38%)	0.002	3(5%)	4(15%)	26(49%)	< 0.0001
TNM stages, I, II	−	−	12		−	−	17	
TNM stages, III, IV	−	−	30		−	−	36	

### Validation of CRC classifiers in the French cohort

The heterogeneity of gut microbiota caused by geographical or ethnical disparities or even technical variations in experimental procedures may interfere with the performance of model and limit its broad application. We hence sought to validate the evaluation performance in an independent group of individuals from France (population B, see Table 1 for patient information), whose gut microbiota OTUs were obtained by shortgun metagenomics sequencing and matched to the marker species using a best-hit approach based on the respective 16S fragments.

In general, population A and B displayed some similarities. Both populations had a similar distribution of gender, whereas CRC patients in both populations were older on average. For the gut microbiome structure, both populations showed an increased ratio of two dominant bacterial divisions, the Bacteroidetes and the Firmicutes, in CRC patients. And both populations showed increased Fusobacteria, Proteobacteria and decreased Actinobacteria in CRC patients [20, 32]. However, big differences also existed. For example, the most distinguishable species in two populations differed. In population A, *Methanosphaera_stadtmanae_DSM_30* and *Blautia_uncultured_Firmicutes_bacterium* seemed to be the most discriminative ones between CRC and controls, while in population B, it was *unclassified Fusobacterium* [1481] that displayed the biggest abundance difference.

As shown in Figure 1B, the overall test performance of all algorithms was decreased in population B compared with those in population A. However, Bayes Net and Random Forest algorithm still achieved AUCs of 0.858 (SD, 0.096) and 0.86 (SD, 0.11), respectively. As the Bayes Net and Random Forest model also maintained high performance of 0.93 (AUC) and 0.94 (AUC) on study population A, we speculated these two models were better models using fecal microbiome to predict CRC, regardless of geographical or cultural disparity or technical variations. We depicted ROC curves for the performance of the two models in population A and B (Figure 1C). When we further analyzed the confusion matrix of Bayes Net (Figure 1E) and Random Forest (Figure 1F) algorithms for population A, we found the false negative rate for Random Forest algorithms (15/46) was higher than that of Bayes Net algorithms (7/46), limited its application as a screening tool. Hence, Bayes Net algorithm outperformed all other classifiers and should be prioritized.

It is worth noting that Simple Logistic and LMT algorithm, two algorithms reached the highest test performance in population A, only displayed limited performance of 0.714 (AUC) and 0.762 (AUC) in population B, respectively (Figure 1D), indicating the performance of different prediction models may vary considerably between populations/races. In term of general applicability, the Bayes Net model appeared to be appropriate models with relatively satisfactory sensitivity, AUCs and false negative rate in both populations. We also listed the overall summary of performance included TPR, FPR, Precision, Recall, F measure and MCC for these four algorithms to better evaluate them (Table 2).

**Table 2:** Overall summary of performance of algorithms

Models	Population A								Population B						
	TPR	FPR	Precision	Recall	F	MCC	AUC		TPR	FPR	Precision	Recall	F	MCC	AUC
**BN**	0.887	0.133	0.888	0.887	0.887	0.745	0.93		0.801	0.255	0.799	0.801	0.798	0.568	0.858
**RF**	0.858	0.237	0.858	0.858	0.853	0.668	0.94		0.773	0.354	0.799	0.773	0.751	0.515	0.86
**SL**	0.922	0.127	0.922	0.922	0.921	0.82	0.975		0.73	0.395	0.737	0.73	0.707	0.402	0.714
**LMT**	0.922	0.127	0.922	0.922	0.921	0.82	0.975		0.773	0.302	0.771	0.773	0.766	0.503	0.762

BN means Bayes Net, RF means Random Forest, SL denotes Simple Logistic. F means F measure. MCC means Matthews correlation coefficient.

### Detection of CRA by Bayes Net model

We further investigated the utility of fecal microbiome for identifying adenoma, which is more difficult to screen compared to CRC but quite necessary for early intervention [33, 34]. Indeed, the overall CRA (colorectal adenoma) AUCs were lower compared with AUCs of CRC in both populations. Consistently, algorithms used gut microbiome from population B showed poorer test performance relative to that from population A. However, in line with the case in CRC, Bayes Net still showed satisfactory test performance of 0.871 AUC (SD, 0.13) in population A (Supplementary Figure S1). Therefore, the fecal microbiome offers new opportunities for non-invasive detection of carcinoma as well as adenoma.

### Unclassified OTUs increased the test performance compared with classified OTUs

**Figure 2:**
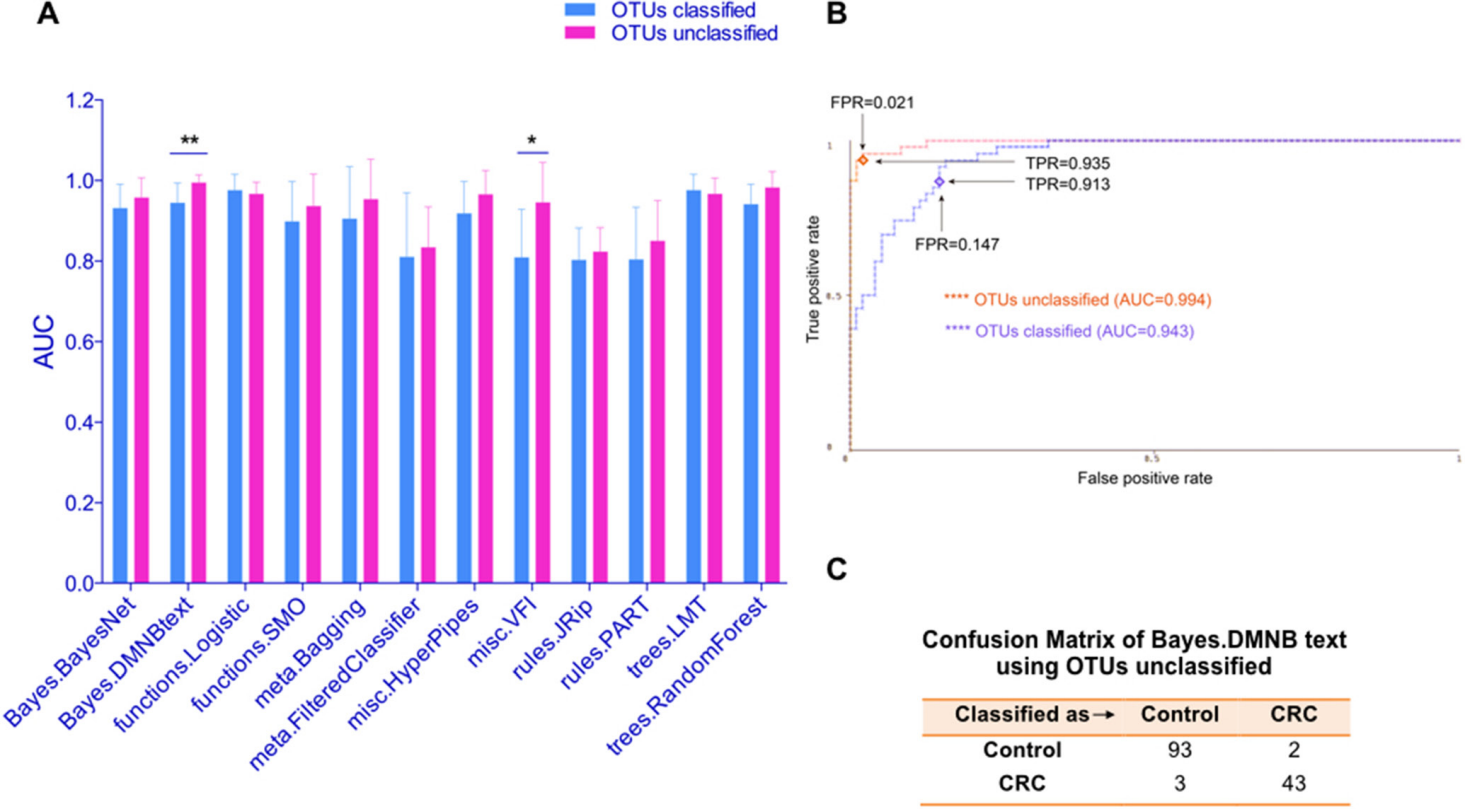
Unclassified OTUs increased the test performance compared with classified OTUs (**A**) The test performance of different models using OTUs dataset classified or not on study population A was displayed using AUC. (**B**) ROC curves show the test performance of the Bayes DMNB text algorithm using OTUs dataset classified or not. TPR (sensitivity) and FPR (false positive rate=1-sensitivity) were also shown. (**C**) Confusion Matrix of Bayes.DMNB text algorithm using OTUs unclassified in population A. *P < 0.05, **P < 0.01.

The pre-processing of 16s sequencing data includes a classification step, which merges different sequencing reads with > 97% similarity into one same species. For example, OTU14, OTU6789 and OTU13450 all link to *Fusobacterium nucleatum*. Interestingly, we found if we use the dataset before merge (i.e. unclassified OTUs), the test performance of almost all models improved (Figure 2A). The most prominent one is the Bayes DMNB text algorithm, the performance of which increased to an AUC of 0.994 (SD, 0.02), corresponding to a relative gain in sensitivity (i.e. TPR) to 0.935 and a decline of false positive rate (FPR) to 0.021 (Figure 2B). Figure 2C showed the confusion matrix of Bayes DMNB text algorithm using unclassified OTUs of population A. This result suggested that the data type (classified or not) also impacted on the optimization of the prediction model.

### Fecal microbiome combined with FOBT moderately improved the prediction ability

**Figure 3:**
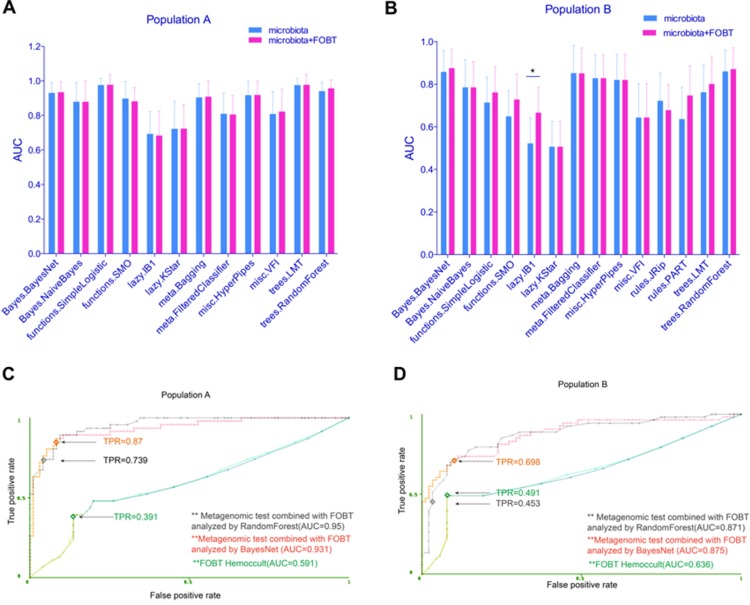
Fecal microbiome combined with FOBT moderately improved the test prediction ability (**A**) The test performance of different classifiers using fecal microbiome combined with FOBT on study population A was displayed with AUC. (**B**) The test performance of different models using fecal microbiome combined with FOBT on study population B was displayed using AUC. (**C** and **D**) ROC curves show the test performance of the Bayes Net and Random Forest models using fecal microbiome in combination with FOBT on study population A and B, respectively. The test performance of FOBT alone was also shown. TPR means true positive rate (sensitivity). *P < 0.05.

Despite its limited sensitivity and specificity, FOBT is the currently standard noninvasive screening tool for CRC. To assess whether FOBT could help improve the test performance of fecal microbiome, we combined FOBT and fecal microbiome information. On this dataset, the test prediction ability of many models showed a slightly improvement, more significantly on study population B (Figure 3A and 3B). For Bayes Net and Random Forest, two algorithms we considered as proper prediction models for CRC, both got a higher AUC after the combination. As shown in Figure 3C, the performance of FOBT alone in population A was just 0.591 AUC, much lower than that of fecal microbiome (0.93 AUC and 0.94 AUC by Bayes Net and Random Forest model, respectively). When we combined FOBT with fecal microbiome, the prediction ability of Bayes Net and Random Forest model increased to an AUC of 0.931 (SD, 0.06) and 0.95 (SD, 0.05), respectively. And the sensitivity reached to 0.87 and 0.739, respectively. These two models showed a similar pattern on study population B (Figure 3D). These data suggested that, in combination with FOBT, fecal microbiome could better predict CRC.

### Interpretation of gut microbial species in the prediction model

Each algorithm has its own default parameters due to the nature of the algorithm itself. However, some quite significantly differently expressed bacteria between CRC and normal controls can be recognized by many different algorithms and used as key parameters to predict. For example, in Chinese population, *Methanosphaera_stadtmanae_DSM_3091* was identified and used by Filtered Classifier, SMO, Logistic, and Naïve Bayes models as key parameters. Another dominant bacterium, *Blautia_uncultured_Firmicutes_bacterium*, was taken by Random Tree, J48 and PART algorithm as a key parameter. In the French population, *unclassified Fusobacterium* [1481] made a second big contribution to the LASSO model to predict CRC. In our analysis, Simple Cart, Rotation Forest, Simple Logistic and Bayes.DMNB text also recognized this bacterium and used it as a default parameter.

**Figure 4:**
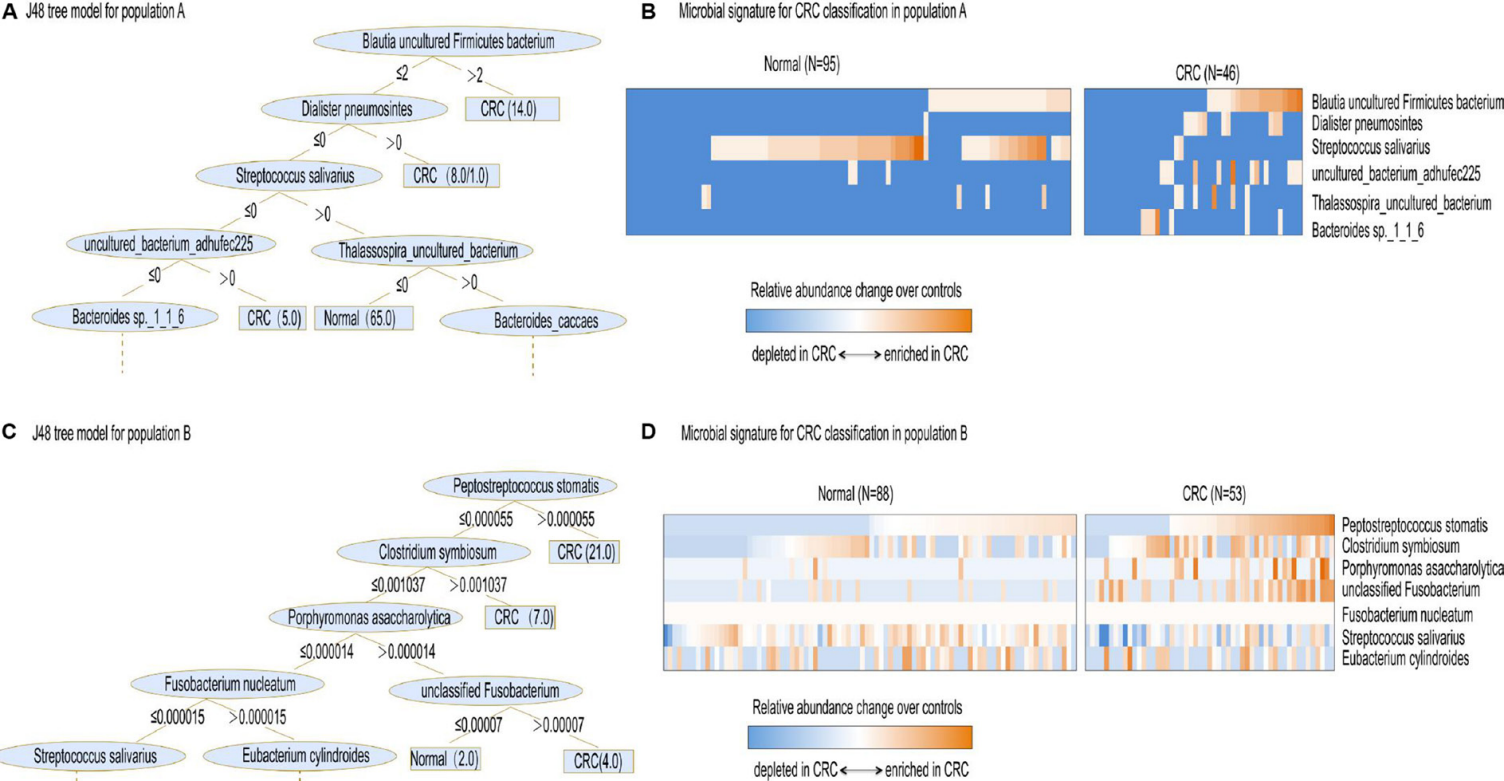
Interpretation of gut microbial species in the prediction model (**A**) The resulting decision tree of J48 model on study population A. Only the leaf nodes at the higher levels are displayed while the rest are indicated by dashes. The leaf nodes that are assigned by one or two numbers where the former and latter indicate the number of correctly and incorrectly classified samples, respectively. (**B**) Relative abundances of 6 gut microbial species, collectively associated with CRC analyzed by J48 model, are displayed as heat map. (**C**) The resulting decision tree of J48 model on study population B. (**D**) Relative abundances of 7 gut microbial species, collectively associated with CRC analyzed by J48 model, are displayed as heat map.

To better show the most discriminative bacterial species that consist of default parameters in many algorithms, we then used J48 algorithm. J48 is used to learn decision trees using quantitative values of attributes. Unlike Bayes Net and Random Forest, which used a multitude of attributes, J48 only analyzed several attributes, making it easy for interpretation of the classification model. As illustrated in Figure 4A, the three most discriminative species in population A were *Blautia uncultured Firmicutes bacterium*, *Dialister pneumosintes* and *Streptococcus salivarius*. In population B, different species consisted of the most CRC-associated gut microbial speices, i.e. *Peptostreptococcus stomatis, Clostridium symbiosum, Porphyromonas asaccharolytica, Fusobacterium nucleatum, unclassified Fusobacterium* and *Streptococcus salivarius* (Figure 4C). Notably, while these two distinct population displayed different CRC-associated fecal microbial species, *Streptococcus salivarius* was identified to make a difference in both populations. Moreover, *Streptococcus salivarius* was more abundant in normal individuals compared with CRC patients in both populations (Figure 4B and 4D).

## DISCUSSION

With the increasing evidence linking gut microbiota and CRC, fecal microbiota has emerged as a promising candidate to non-invasively screen for CRC. However, it is still unclear which classification models should be more applicable in fecal microbiota-based CRC prediction. In this study, we confirmed that fecal microbiota could be used as a noninvasive CRC screening tool, being more accurate than FOBT. By evaluating supervised machine-learning algorithms in a comprehensive manner, we found different algorithms had quite different prediction performance using fecal microbiota. Population disparities, variation in experimental procedures and different data types also played a role in the optimization of the prediction model, while Bayes Net turned out to be a good model generally.

An exciting and potentially far-reaching development in computer science is the invention and application of methods of machine learning (ML). It enables a computer program to automatically analyze a large body of data and decide what information is most relevant. It was widely used in studies as diverse as methylated DNA patterns linked to genetic disorders [35]and Alzheimer's disease (AD) diagnosis using imaging data [28]. In this work, we tested all algorithms available in machine learning and found Bayes Net algorithm provided better performance as assessed from ROC curve analysis and false negative rate in both populations. Notably, Bayes Net algorithm was able to classify colorectal adenomas from normal controls. This has considerable importance because screening for early-stage colorectal cancer depends on the ability to detect early pathologic changes [19].

As mentioned above, another three independent studies suggested the potential of fecal microbiome as a non-invasive CRC screening tool—using three different models. The data information of our population B just extracted from the study of Georg Zeller [20], who employed LASSO classifier and reached a 0.84 AUC. Obviously, Bayes Net showed a moderate superiority to LASSO classifier, with the performance of 0.858 AUC based on the same dataset. P. Zackular [19] also used Bayesian methods to classify CRC patient and normal controls. But he only got a test performance of 0.798 AUC, much lower than our 0.93 AUC performance. Besides population differences, the data type differences should be accountable for the limited accuracy. As he only used 6 OTUs at the family rank, we used all OTUs at the species rank. Therefore, data type also affects the performance of algorithms and fecal microbiota dataset with the lower ranks was recommended to predict CRC. Consistent with our result, the third study [21], also reported a good prediction of Random Forest algorithm to predict CRC (AUC=0.96). But he did not report the false negative rate. While in our study, we found that despite Random Forest had a satisfactory AUC value, it also had a high false negative rate, which greatly weakened its application in screening CRC.

Another important finding is that a predominant oral cavity commensal, *Streptococcus salivarius*, was selected as a significant microbe to classify CRC patients and tumor-free controls by J48 algorithm in two independent groups as well as by LASSO model in Zeller’ study. This consistency highlighted the potential beneficial effect of *Streptococcus salivarius*. Notably, some strains *of S. salivarius* are found to produce BLIS (Bacteriocin-like Inhibitory Substances), which is an antimicrobial peptide and are being trialed for their use as a probiotic in the prevention of oral infections [36–39]. Our identification of the oral commensal *Streptococcus salivarius* to be much more depleted in CRC patient feces implicated it could be a promising probiotic agent used for treatment of CRC.

Taken together, our results suggest that Bayes Net algorithm displayed quite good performance in both populations and could be used in future studies. Unclassified OTUs were better inputs than classified OTUs.

## MATERIALS AND METHODS

### Sample collection for study population A

Participants of population A were selected from consecutive patients who had undergone colonoscopy or colorectal carcinoma surgery in the Renji Hospital between 1 January 2012 and 30 July 2012. The inclusion criteria for participants to enter the study were the same as our previous study [32]. In brief, patients were over 50 years of age, had a normal bowel frequency and patients underwent colonoscopies with adequate withdrawal time by well-trained gastroenterologists using standard colonoscopy equipment. Patients with cirrhotic or portal hypertension gastropathy; uncontrollable diabetes mellitus or hypertension; severe cardiac, liver, pulmonary, renal, hematologic, or rheumatologic disorders; or CRC-related conditions, such as familial adenomatous polyposis, hereditary nonpolyposis colorectal cancer, ulcerative colitis, or Crohn disease, were excluded from the study. None of the patients had received systemic or oral topical corticosteroids; antibiotics, aspirin, other nonsteroidal anti-inflammatory drugs, or health products that regulate intestinal microbiota within 6 month before enrollment, and none were currently undergoing systemic cancer chemotherapy or receiving radiation. Patients with CRC or CRA (colorectal adenoma) confirmed by both colonoscopy and pathological examination were included in the CRC group or CRA group, respectively. Patients without obvious abnormalities were enrolled in the NC (Negative control) group. Finally, 52 cases in the NC group, 47 patients in the CRA group and 42 patients in the CRC group were enrolled. All subjects were asked to provide fresh stool samples, which were immediately stored at −80°C for further analysis. The samples were collected and preserved according to our previous study [40]. All procedures were undertaken in accordance with the Declaration of Helsinki. The Ethics Committees in the Renji Hospital at each participating center approved the study protocol. Informed consent was obtained from all of the subjects. An independent data and safety committee monitored the trial and reviewed the results.

### Acquisition of published fecal microbiome data

In parallel, another fecal microbiome dataset [20] was used as study population B to validate our results (Table 1). Mainly, 141 subjects that consisted of 61 normal individuals, 27 patients with small adenomas and 53 patients with colorectal cancers were included. When we compared the prediction ability for different algorithms between CRC group and tumor-free controls, 27 patients with small adenomas together with 61 normal individuals were used as the control group. The primary dataset B consisted of 156 participants, with 15 patients with large adenomas. The author excluded these 15 patients in their analysis. We followed the selection criteria and used the remained 141 participants as our validation cohort.

### DNA extraction and 16S rRNA gene sequencing

Microbial genomic DNA was extracted from frozen fecal samples using the E.Z.N.A. Stool DNA Kit (Omega Bio-Tek, Inc., Norcross, GA, USA). The DNA concentration was determined and tested by 1% agarose gel electrophoresis. All DNA samples were stored at –20°C for subsequent PCR analysis. DNA purification was performed by using the AxyPrep DNA Gel Extraction Kit (catalog no. AP-GX-50; Axygen), and the TBS-380 Fluorometer was used for quantitative analysis. The V1∼V3 hypervariable regions of the 16S rRNA gene(27F 5′-AGAGTTTGATCCTGGCTCAG-3′, 533R 5′-TTACCGC GGCTGCTGGCAC-3′) incorporating the FLX Titanium adaptors and a sample barcode sequence from each sample were amplified using a 2-step PCR strategy(Takara Bio Inc).After the PCR reaction, electrophoresis was immediately performed to isolate the enriched V1-V3 region DNA fragments from the reaction mixture. All of the products were harvested by using a gel extraction kit (OMEGA Bio-tek) according to the manufacturer's instructions. Then the samples were pyrosequenced by using a Roche 454 GS FLX, in accordance with the manufacturer's instructions. The gross sequencing data were arranged by the primer tags [41]. The sequences were binned into each sample according their barcodes and forward primers. A total of 2905689 sequences were obtained and analyzed using MOTHUR software package. Briefly, 1) the reverse primer and adaptor at the end of the sequences, polybasic N, poly A/T tail and low quality bases were removed; 2) After that, barcodes and forward primers of the sequences in 1) were removed; 3) sequences that were < 200 base pairs, or contained ambiguous bases or average sequence quality was less than 25 were abandoned. After optimization, we obtained a total of 2296326 sequences for 141 samples with an average length of 437bp per sample. Sequences were clustered into operational taxonomic units (OTU) at a 97% similarity cutoff and the relative abundance was calculated for OTUs in each sample. Taxonomy information was obtained for each OTU sample by cross-referencing the SILVA database.

### Machine learning classifiers

All classification and analyses were performed on the Weka (3.6.13 version) program package. Weka is open source software, which provides a general-purpose environment for automatic classification, regression, clustering, association and feature selection-common data mining problems in bioinformatics research. It contains an extensive collection of different machine learning algorithms (e.g. decision trees, rule sets, linear discriminants). We tested all available classifiers (algorithms) in Weka. Here we briefly introduce the characteristics of algorithms with higher performance in our work. These models have also been reported to evolve to largely dominate other many data mining approaches that have been explored. They are 1) Bayes Net algorithm is a formal graphical language for representing the joint probability distribution over a set of random variables [42]. Bayesian networks are convenient in that they provide an intuitive and compact representation of the joint distribution of this set of variables, and they expose ways to utilize their dependencies to perform statistical inference. 2) Random Forest algorithm is a form of multiple decision trees that are built partly randomly. After a multitude of trees are generated, each tree in the forest gives a classification or votes for a class and the most popular class gives the final classification. The main advantage of this method is that it is fast while capable of handling of large input variables without over-fitting at the same time [43]. 3) J48 algorithm is the Weka version of the C4.5 algorithm. It creates a tree data structure that can be used to classify new instances. First, the pair (attribute, value) that optimizes a criterion is used to split the data into two branches. Then for each branch, if it is pure or if it contains less than a predefine number of data, the branch stopped growing, else another split is decided on the basis of the same algorithm [44]. These algorithms classify input data into separable classes based on defined attributes, which in ML are generally known as “features”. In our study, the diagnosis feature was used as “class”. And different OTUs or FOBT were used as attributes. Default parameters were used for model generation on these datasets. The main steps of the algorithms are 1) preparing data for input into classifier training, 2) training and testing the classifier, and 3) evaluating its performance.

### Data preprocessing

Analyses of patient-level characteristics across the 3 clinical groups utilized Pearson χ^2^ test for categorical data and one-way ANOVA for continuous variables. *P* < 0.05 was considered statistically significant.

For gut microbiome analysis, only OTUs in the species level were used. In population A, a total of 1171 classified OTUs were selected as “attributes” and in population B, 783 OTUs were included. When we use unclassified OTUs to compute in population A, the OTUs number is 7730. When we analyzed the performance between NC group and CRC group, the CRA patients were considered and included into the NC group. The same data preprocess were done in the French population (population B) (Table 1). All files were saved as ARFF or CSV file formats.

### Training and testing the classifiers

In the second step, classifiers were trained using data predefined into groups of interest (normal, CRA and CRC patients in our study) to get a “decision function”, which best distinguishes between different classes in the test datasets. The training dataset and test dataset in this study was produced by 10-fold cross-validation. Cross-validation randomly splits the original sample into 10 subsamples, 9 of which are used as training data and the remaining one is retained as the validation data for testing the classifiers. This process is repeated 10 times, with each of the 10 subsamples used exactly once as the testing data. Then the 10 results were averaged to produce a single estimation. This is a standard procedure in machine learning to reduce the variation of data selection.

### Evaluation of classifiers

The performance of the algorithms was mainly displayed using receiver operating characteristic (ROC) curve analysis. As ROC curve analysis is regarded as one of the reliable and best approach for performance characterization, the ROC curve and area under ROC curve (AUC) values are widely employed for assessing the discriminatory power of virtual screens. Standard deviation was used to assess the variability of the classifier performance.

One key issue that needs to be taken into account while using machine learning is whether the dataset is imbalanced or not. Standard algorithms presume equal weighing for all groups, which is always not true in reality. One way to abrogate this issue and minimize the misclassification errors was to use the cost-sensitive classifiers. In the present study, Weka uses a confusion matrix consisting of four sections: True positives (TP) for correctly classified as CRC; False Positive (FP) for normal controls classified as CRC; True Negatives (TN) normal controls classified as normal controls and false negatives (FN) for CRC incorrectly classified as normal controls. Weka also presents Precision, Recall and F measure for all classifiers. Precision is referred to as positive predictive value. It can be calculated as (TP/TP+FP). Recall (TP/TP+FN) also calls sensitivity. It relates to the test's ability to identify positive results. F measure (F1 score) is the harmonic mean of precision and recall. In addition, we also calculated Matthews correlation coefficient (MCC) to judge performance of some algorithms that performed well in our study. MCC is generally regarded as a balanced measure that can be used even if the classes are of very different sizes. Its value ranges from −1 to +1. A coefficient of +1 represents a perfect prediction, 0 no better than random prediction and −1 indicates total disagreement between prediction and observation. As our study groups are tolerably balanced (52 NC, 47 CRA and 42 CRC in population A and 61 NC, 27 CRA and 53 CRC in population B), and the negative controls even outnumber the positive CRC patients, we just evaluated the performance of algorithms judging from the confusion matrix besides AUC. We list the Precision, Recall, F measure, MCC and even confusion matrix for some of algorithms that work wonderful in our work.

## SUPPLEMENTARY FIGURE AND TABLES



## References

[1] Jemal A, Center MM, DeSantis C, Ward EM (2010). Global patterns of cancer incidence and mortality rates and trends. Cancer Epidemiol Biomarkers Prev.

[2] Center MM, Jemal A, Smith RA, Ward E (2009). Worldwide variations in colorectal cancer. CA Cancer J Clin.

[3] Haug U, Knudsen AB, Lansdorp-Vogelaar I, Kuntz KM (2015). Development of new non-invasive tests for colorectal cancer screening: the relevance of information on adenoma detection. Int J Cancer.

[4] Parente F, Marino B, Ilardo A, Fracasso P, Zullo A, Hassan C, Moretti R, Cremaschini M, Ardizzoia A, Saracino I, Perna F, Vaira D (2012). A combination of faecal tests for the detection of colon cancer: a new strategy for an appropriate selection of referrals to colonoscopy? A prospective multicentre Italian study. Eur J Gastroenterol Hepatol.

[5] Dickinson BT, Kisiel J, Ahlquist DA, Grady WM (2015). Molecular markers for colorectal cancer screening. Gut.

[6] Faivre J, Dancourt V, Lejeune C, Tazi MA, Lamour J, Gerard D, Dassonville F, Bonithon-Kopp C (2004). Reduction in colorectal cancer mortality by fecal occult blood screening in a French controlled study. Gastroenterology.

[7] Hardcastle JD, Chamberlain JO, Robinson MH, Moss SM, Amar SS, Balfour TW, James PD, Mangham CM (1996). Randomised controlled trial of faecal-occult-blood screening for colorectal cancer. Lancet.

[8] Scholefield JH, Moss S, Sufi F, Mangham CM, Hardcastle JD (2002). Effect of faecal occult blood screening on mortality from colorectal cancer: results from a randomised controlled trial. Gut.

[9] Mandel JS, Bond JH, Church TR, Snover DC, Bradley GM, Schuman LM, Ederer F (1993). Reducing mortality from colorectal cancer by screening for fecal occult blood. Minnesota Colon Cancer Control Study. N Engl J Med.

[10] Levin B, Lieberman DA, McFarland B, Andrews KS, Brooks D, Bond J, Dash C, Giardiello FM, Glick S, Johnson D, Johnson CD, Levin TR, Pickhardt PJ (2008). Screening and surveillance for the early detection of colorectal cancer and adenomatous polyps, 2008: a joint guideline from the American Cancer Society, the US Multi-Society Task Force on Colorectal Cancer, and the American College of Radiology. Gastroenterology.

[11] Ransohoff DF, Pignone M (2001). The effect of fecal occult-blood screening on the incidence of colorectal cancer. N Engl J Med.

[12] Zou H, Taylor WR, Harrington JJ, Hussain FT, Cao X, Loprinzi CL, Levine TR, Rex DK, Ahnen D, Knigge KL, Lance P, Jiang X, Smith DI (2009). High detection rates of colorectal neoplasia by stool DNA testing with a novel digital melt curve assay. Gastroenterology.

[13] Wang T, Cai G, Qiu Y, Fei N, Zhang M, Pang X, Jia W, Cai S, Zhao L (2012). Structural segregation of gut microbiota between colorectal cancer patients and healthy volunteers. Isme j.

[14] Ahn J, Sinha R, Pei Z, Dominianni C, Wu J, Shi J, Goedert JJ, Hayes RB, Yang L (2013). Human gut microbiome and risk for colorectal cancer. Journal of the National Cancer Institute.

[15] Mima K, Nishihara R, Qian ZR, Cao Y, Sukawa Y, Nowak JA, Yang J, Dou R, Masugi Y, Song M, Kostic AD, Giannakis M, Bullman S (2015). Fusobacterium nucleatum in colorectal carcinoma tissue and patient prognosis. Gut.

[16] Wong SH, Kwong TN, Chow TC, Luk AK, Dai RZ, Nakatsu G, Lam TY, Zhang L, Wu JC, Chan FK, Ng SS, Wong MC, Ng SC (2016). Quantitation of faecal Fusobacterium improves faecal immunochemical test in detecting advanced colorectal neoplasia. Gut.

[17] Goodwin AC, Destefano Shields CE, Wu S, Huso DL, Wu X, Murray-Stewart TR, Hacker-Prietz A, Rabizadeh S, Woster PM, Sears CL, Casero RA (2011). Polyamine catabolism contributes to enterotoxigenic Bacteroides fragilis-induced colon tumorigenesis. Proceedings of the National Academy of Sciences of the United States of America.

[18] Arthur JC, Perez-Chanona E, Muhlbauer M, Tomkovich S, Uronis JM, Fan TJ, Campbell BJ, Abujamel T, Dogan B, Rogers AB, Rhodes JM, Stintzi A, Simpson KW (2012). Intestinal inflammation targets cancer-inducing activity of the microbiota. Science.

[19] Zackular JP, Rogers MAM, Ruffin MT, Schloss PD (2014). The Human Gut Microbiome as a Screening Tool for Colorectal Cancer. Cancer Prevention Research.

[20] Zeller G, Tap J, Voigt AY, Sunagawa S, Kultima JR, Costea PI, Amiot A, Bohm J, Brunetti F, Habermann N, Hercog R, Koch M, Luciani A (2014). Potential of fecal microbiota for early-stage detection of colorectal cancer. Mol Syst Biol.

[21] Feng Q, Liang S, Jia H, Stadlmayr A, Tang L, Lan Z, Zhang D, Xia H, Xu X, Jie Z, Su L, Li X, Li X (2015). Gut microbiome development along the colorectal adenoma-carcinoma sequence. Nat Commun.

[22] Nassif H, Wu Y, Page D, Burnside E (2012). Logical Differential Prediction Bayes Net, improving breast cancer diagnosis for older women. AMIA Annu Symp Proc.

[23] Gevaert O, De Smet F, Timmerman D, Moreau Y, De Moor B (2006). Predicting the prognosis of breast cancer by integrating clinical and microarray data with Bayesian networks. Bioinformatics.

[24] Xue X, Zeng N, Gao Z, Du MQ (2015). Diffuse large B-cell lymphoma: sub-classification by massive parallel quantitative RT-PCR. Lab Invest.

[25] Shabbeer A, Cowan LS, Ozcaglar C, Rastogi N, Vandenberg SL, Yener B, Bennett KP (2012). TB-Lineage: an online tool for classification and analysis of strains of Mycobacterium tuberculosis complex. Infect Genet Evol.

[26] Habibi S, Ahmadi M, Alizadeh S (2015). Type 2 Diabetes Mellitus Screening and Risk Factors Using Decision Tree: Results of Data Mining. Glob J Health Sci.

[27] Kourou K, Exarchos TP, Exarchos KP, Karamouzis MV, Fotiadis DI (2015). Machine learning applications in cancer prognosis and prediction. Comput Struct Biotechnol J.

[28] Lebedev AV, Westman E, Van Westen GJ, Kramberger MG, Lundervold A, Aarsland D, Soininen H, Kloszewska I, Mecocci P, Tsolaki M, Vellas B, Lovestone S, Simmons A (2014). Alzheimer's Disease Neuroimaging I and the AddNeuroMed c. Random Forest ensembles for detection and prediction of Alzheimer's disease with a good between-cohort robustness. Neuroimage Clin.

[29] Takahashi N, Guo J, Nishi T (2008). Global convergence of SMO algorithm for support vector regression. IEEE Trans Neural Netw.

[30] Wundersitz DW, Josman C, Gupta R, Netto KJ, Gastin PB, Robertson S (2015). Classification of team sport activities using a single wearable tracking device. J Biomech.

[31] Li J, Gramatica P (2010). Classification and virtual screening of androgen receptor antagonists. J Chem Inf Model.

[32] Yu YN, Yu TC, Zhao HJ, Sun TT, Chen HM, Chen HY, An HF, Weng YR, Yu J, Li M, Qin WX, Ma X, Shen N (2015). Berberine may rescue Fusobacterium nucleatum-induced colorectal tumorigenesis by modulating the tumor microenvironment. Oncotarget.

[33] Souverijn JH (2014). Multitarget stool DNA testing for colorectal-cancer screening. N Engl J Med.

[34] Brenner H, Kloor M, Pox CP (2014). Colorectal cancer. Lancet.

[35] Ghorbani M, Taylor SJ, Pook MA, Payne A (2013). Comparative (computational) analysis of the DNA methylation status of trinucleotide repeat expansion diseases. J Nucleic Acids.

[36] Di Pierro F, Adami T, Rapacioli G, Giardini N, Streitberger C (2013). Clinical evaluation of the oral probiotic Streptococcus salivarius K12 in the prevention of recurrent pharyngitis and/or tonsillitis caused by Streptococcus pyogenes in adults. Expert Opin Biol Ther.

[37] Di Pierro F, Colombo M, Zanvit A, Risso P, Rottoli AS (2014). Use of Streptococcus salivarius K12 in the prevention of streptococcal and viral pharyngotonsillitis in children. Drug Healthc Patient Saf.

[38] Di Pierro F, Di Pasquale D, Di Cicco M (2015). Oral use of Streptococcus salivarius K12 in children with secretory otitis media: preliminary results of a pilot, uncontrolled study. Int J Gen Med.

[39] Di Pierro F, Zanvit A, Nobili P, Risso P, Fornaini C (2015). Cariogram outcome after 90 days of oral treatment with Streptococcus salivarius M18 in children at high risk for dental caries: results of a randomized, controlled study. Clin Cosmet Investig Dent.

[40] Chen HM, Yu YN, Wang JL, Lin YW, Kong X, Yang CQ, Yang L, Liu ZJ, Yuan YZ, Liu F, Wu JX, Zhong L, Fang DC (2013). Decreased dietary fiber intake and structural alteration of gut microbiota in patients with advanced colorectal adenoma. Am J Clin Nutr.

[41] Meyer M, Stenzel U, Hofreiter M (2008). Parallel tagged sequencing on the 454 platform. Nat Protoc.

[42] Libbrecht MW, Noble WS (2015). Machine learning applications in genetics and genomics. Nat Rev Genet.

[43] Periwal V, Kishtapuram S, Open Source Drug Discovery C, Scaria V (2012). Computational models for in-vitro anti-tubercular activity of molecules based on high-throughput chemical biology screening datasets. BMC Pharmacol.

[44] Aze J, Sola C, Zhang J, Lafosse-Marin F, Yasmin M, Siddiqui R, Kremer K, van Soolingen D, Refregier G (2015). Genomics and Machine Learning for Taxonomy Consensus: The Mycobacterium tuberculosis Complex Paradigm. PloS one.

