# Supplementary material for: Systematic evaluation of supervised classifiers for fecal microbiota-based prediction of colorectal cancer
Source: Oncotarget. 2017 Jan 4;8(6):9546–56. doi: 10.18632/oncotarget.14488 (PMC5354752; doi:10.18632/oncotarget.14488)
Supplement: Supplementary file 1 [file oncotarget-08-9546-s001.pdf]

Systematic evaluation of supervised classifiers for fecal microbiota-based prediction of colorectal cancer

Supplementary Materials

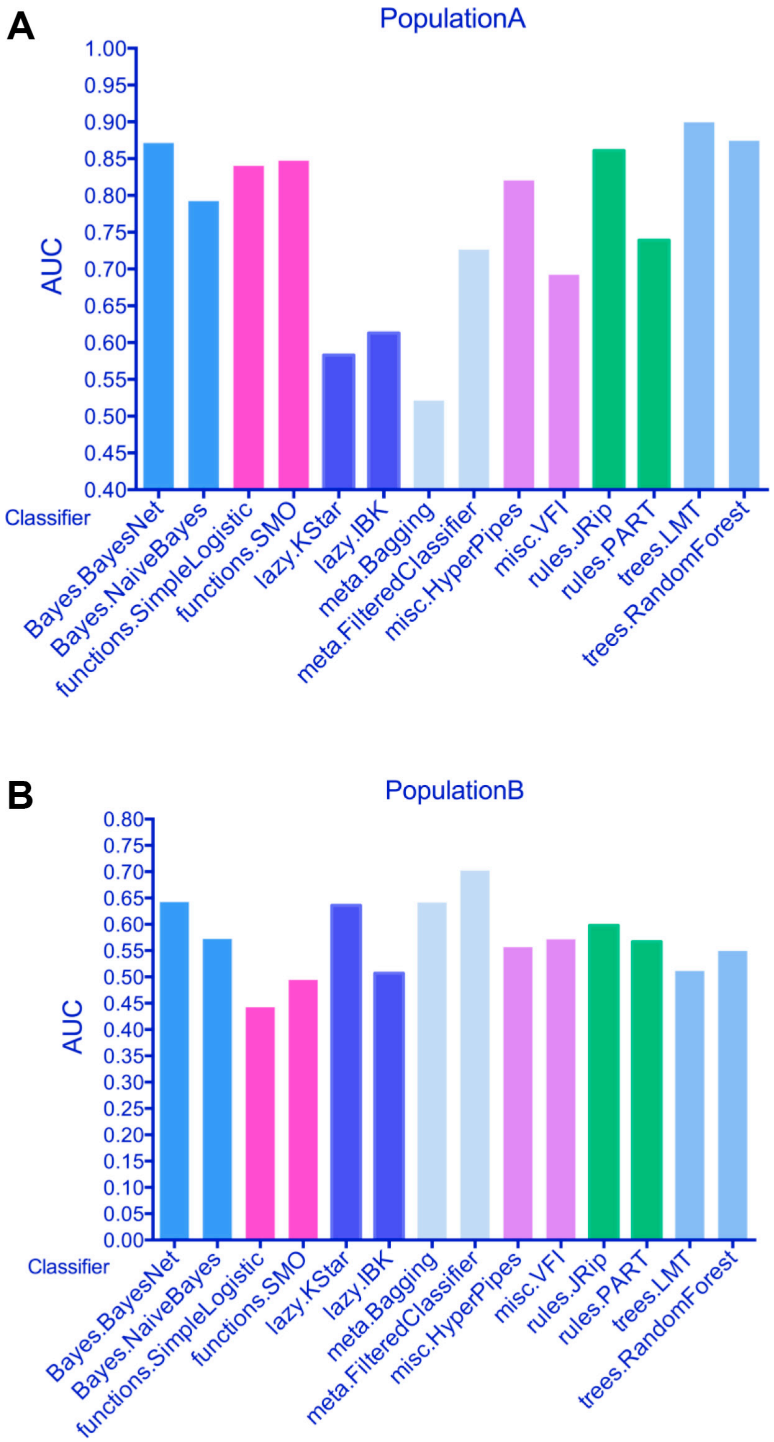

Supplementary Figure S1: The CRA prediction accuracy based on fecal microbiome varied among different classifiers and populations. (**A**) The test accuracy of different models using fecal microbiome on study population A was displayed using area under roc curve (AUC). (**B**) The test accuracy of different models using fecal microbiome on study population B was displayed using AUC.

Supplementary Table S1: The performance of different algorithms in population A

Classifier	Weighted Avg	CRA	CRC	NC
Bayes.Bayes Net	0.888	0.871	0.937	0.859
Bayes.Naive Bayes	0.847	0.792	0.908	0.839
Bayes.Naive Bayes Multinomial Text	0.472	0.459	0.461	0.495
Bayes.Naive Bayes Updateable	0.847	0.792	0.908	0.839
functions.Logistic	0.862	0.84	0.9	0.846
functions.Simple Logistic	0.909	0.892	0.959	0.878
functions.SMO	0.87	0.847	0.923	0.841
lazy.IBk	0.663	0.613	0.772	0.608
lazy.KStar	0.581	0.583	0.639	0.524
lazy.WL	0.696	0.631	0.811	0.649
meta.AdaBoostM1	0.774	0.739	0.868	0.72
meta.Attribute Selected Classifier	0.762	0.807	0.828	0.661
meta.Bagging	0.504	0.521	0.493	0.501
meta.Classification Via Clustering	0.531	0.538	0.543	0.515
meta.Classification Via Regression	0.472	0.459	0.461	0.495
meta.CV Parameter Selection	0.472	0.459	0.461	0.495
meta.Dagging	0.855	0.848	0.931	0.79
meta.Decorate	0.855	0.87	0.899	0.855
meta.END	0.867	0.893	0.904	0.81
meta.Filtered Classifier	0.709	0.726	0.753	0.653
meta.Grading	0.5	0.5	0.5	0.5
meta.Iterative Classifier Optimizer	0.875	0.837	0.947	0.842
meta.Logit Boost	0.879	0.845	0.951	0.844
meta.Multi BoostAB	0.76	0.756	0.86	0.67
meta.MultiClass Classifier	0.866	0.831	0.901	0.865
meta.Multi Scheme	0.472	0.459	0.461	0.495
meta.Random Committee	0.8	0.773	0.923	0.711
meta.Randomizable Filtered Classifier	0.615	0.59	0.628	0.626
meta.Random SubSpace	0.87	0.882	0.91	0.821
meta.Stacking	0.472	0.459	0.461	0.495
meta.Vote	0.472	0.459	0.461	0.495
misc.HyperPipes	0.85	0.82	0.919	0.814
misc.VFI	0.719	0.692	0.798	0.67
misc.Input Mapped Classifier	0.472	0.459	0.461	0.495
rules.Conjunctive Rule	0.495	0.495	0.491	0.5
rules.Decision Table	0.791	0.77	0.799	0.803
rules.JRip	0.803	0.861	0.804	0.749
rules.OneR	0.5	0.5	0.5	0.5
rules.NNge	0.737	0.723	0.833	0.661
rules.PART	0.723	0.739	0.773	0.664
rules.ZeroR	0.472	0.459	0.461	0.495
trees.BFTree	0.472	0.459	0.461	0.495
trees.DecisionStump	0.655	0.581	0.757	0.626
trees.FT	0.905	0.887	0.946	0.884
trees.Hoeffding Tree	0.705	0.604	0.829	0.68
trees.J48	0.722	0.768	0.765	0.642
trees.J48 graft	0.73	0.702	0.788	0.702
trees.LAD Tree	0.861	0.86	0.935	0.794
trees.LMT	0.905	0.899	0.948	0.87
trees.Random Forest	0.887	0.874	0.945	0.846
trees.RandomTree	0.613	0.567	0.7	0.574
trees.REP Tree	0.472	0.459	0.461	0.495
trees.Simple Cart	0.472	0.459	0.461	0.495

Supplementary Table S2: The performance of different algorithms in population B

Classifier	Weighted Avg	CRA	CRC	NC
Bayes.Bayes Net	0.723	0.642	0.807	0.685
Bayes.Naive Bayes	0.685	0.572	0.77	0.662
Bayes.Naive Bayes Multinomial Text	0.472	0.45	0.471	0.481
Bayes.Naive Bayes Updateable	0.685	0.572	0.77	0.662
functions.Logistic	0.562	0.457	0.557	0.612
functions.Multilayer Perceptron	0.698	0.553	0.7	0.7
functions.Simple Logistic	0.612	0.442	0.734	0.582
functions.SMO	0.617	0.494	0.727	0.576
lazy.IBk	0.576	0.507	0.591	0.593
lazy.KStar	0.648	0.535	0.679	0.671
lazy.LWL	0.648	0.535	0.679	0.671
meta.AdaBoostM1	0.615	0.444	0.702	0.615
meta.Attribute Selected Classifier	0.725	0.672	0.803	0.682
meta.Bagging	0.737	0.641	0.834	0.696
meta.Classification Via Clustering	0.504	0.5	0.49	0.518
meta.Classification Via Regression	0.688	0.618	0.796	0.624
meta.CV Parameter Selection	0.472	0.45	0.471	0.481
meta.Dagging	0.732	0.605	0.778	0.749
meta.Decorate	0.716	0.574	0.825	0.685
meta.END	0.686	0.492	0.787	0.684
meta.Filtered Classifier	0.656	0.702	0.616	0.798
meta.Grading	0.5	0.5	0.5	0.5
meta.Iterative Classifier Optimizer	0.587	0.425	0.716	0.546
meta.Logit Boost	0.639	0.476	0.735	0.628
meta.MultiBoostAB	0.647	0.471	0.726	0.658
meta.Multi Class Classifier	0.582	0.493	0.607	0.599
meta.Multi Class Classifier Updateable	0.638	0.503	0.716	0.631
meta.Multi Scheme	0.472	0.45	0.471	0.481
meta.Random Committee	0.694	0.519	0.813	0.668
meta.Randomizable Filtered Classifier	0.514	0.427	0.544	0.526
meta.Random SubSpace	0.709	0.604	0.81	0.667
meta.Stacking	0.472	0.45	0.471	0.481
meta.Vote	0.472	0.45	0.471	0.481
misc.HyperPipes	0.699	0.556	0.841	0.64
misc.Input Mapped Classifier	0.472	0.45	0.471	0.481
misc.VFI	0.641	0.571	0.71	0.611
rules.Conjunctive Rule	0.489	0.468	0.486	0.5
rules.Decision Table	0.632	0.558	0.703	0.603
rules.JRip	0.703	0.598	0.77	0.691
rules.NNge	0.665	0.547	0.726	0.663
rules.OneR	0.524	0.553	0.495	0.535
rules.PART	0.673	0.567	0.762	0.641
rules.Ridor	0.5	0.5	0.5	0.5
rules.ZeroR	0.472	0.45	0.471	0.481
trees.BFTree	0.58	0.536	0.616	0.568
trees.Decision Stump	0.558	0.526	0.591	0.543
trees.FT	0.647	0.583	0.724	0.609
trees.HoeffdingTree	0.67	0.596	0.759	0.626
trees.J48	0.657	0.573	0.752	0.612
trees.J48graft	0.525	0.512	0.59	0.475
trees.LAD Tree	0.629	0.654	0.642	0.608
trees.LMT	0.65	0.511	0.746	0.629
trees.Random Forest	0.736	0.549	0.846	0.724
trees.Random Tree	0.546	0.471	0.554	0.572
trees.REPTree	0.595	0.555	0.639	0.576
